# Radiofrequency Ablation in the Management of Advanced Stage Thymomas: A Case Report on a Novel Multidisciplinary Therapeutic Approach

**DOI:** 10.1155/2014/697480

**Published:** 2014-12-10

**Authors:** Panagiotis Paliogiannis, Carlo Pala, Renato Versace, Claudio Pusceddu

**Affiliations:** ^1^Surgical Pathology, Department of Surgical, Microsurgical and Medical Sciences, University of Sassari, Viale San Pietro 43B, 07100 Sassari, Italy; ^2^Department of Thoracic Surgery, Oncological Hospital of Cagliari, Via E. Jenner 1, 09100 Cagliari, Italy; ^3^Department of Radio-Oncology, Oncological Hospital of Cagliari, Via E. Jenner 1, 09100 Cagliari, Italy

## Abstract

We describe in this report a case of successful radiofrequency ablation of an unresectable stage III-type B3 thymoma, and we discuss the role of this novel approach in the management of patients with advanced stage thymoma. The patient, a 59-year-old Caucasian male underwent neoadjuvant chemotherapy with only a slight reduction of the mass. Subsequently, an explorative sternotomy and debulking were performed; before closing the thorax, radiofrequency ablation of the residual tumor was carried out and a partial necrosis of the mass was achieved. A further percutaneous radiofrequency ablation was performed subsequently, obtaining complete necrosis of the lesion. Successively, the patient underwent adjuvant radiotherapy. As a result of this multidisciplinary treatment, complete and stable response was obtained. It is hard to say which of the single treatments had the major impact on cure; nevertheless, the results obtained suggest that radiofrequency ablation must be taken into account for the treatment of advanced stage thymomas, and its effectiveness must be further assessed in future studies.

## 1. Introduction

Thymomas are rare tumors of the thymus gland, accounting approximately for 0.15 per 100.000 person-years in the United States [1]. Early stage thymomas are well managed with surgery or multidisciplinary treatments, while the management of advanced stage thymomas is often demanding, especially of those unresectable, recurrent, or histologically classified as B3 according to the World Health Organization (WHO) classification [1]. Ablative treatments are sporadically reported in the treatment of advanced stage thymomas, consisting exclusively in cryoablation methods [2, 3]. We describe in this report a case of successful radiofrequency ablation (RFA) of an unresectable stage III-type B3 tumor, and we discuss the usefulness of this novel approach in the management of patients with advanced stage thymoma.

## 2. Case Report

**Figure 1:**
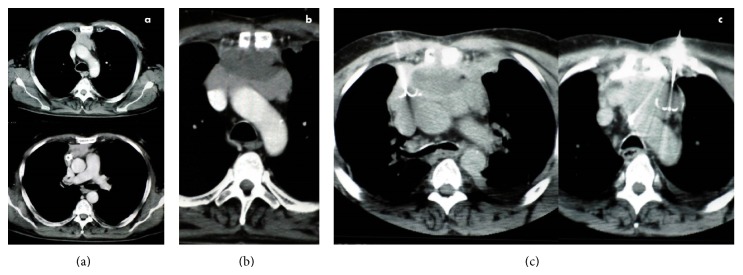
CT scans depicting the preoperative aspect of the lesion (a), the necrosis obtained after surgery and RFA (b), and the positions of the electrodes during percutaneous RFA performed after surgery (c).

A 59-year-old Caucasian male with a stage III-type B3 thymoma was referred to the Businco Oncological Hospital of Cagliari, Italy, in 2006. The patient underwent four cycles of cisplatin, doxorubicin, vincristine, and cyclophosphamide, with only a slight reduction of the mass (Figure 1(a)).

**Figure 2:**
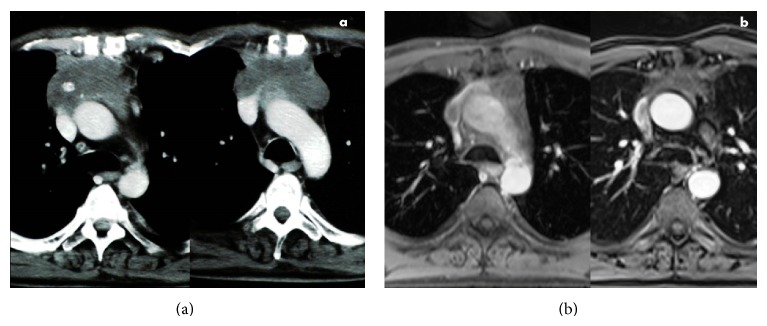
CT scans showing the local conditions of the patient one month after percutaneous RFA (a) and NMR depicting complete response 36 months after treatment (b).

The surgeons performed an explorative sternotomy and, once the unresectability of the lesion was confirmed, a debulking. Before closing the thorax, an RFA of the residual tumor was performed with a needle monopolar electrode, as we previously described, positioned in the centre of the lesion, and a partial necrosis of the mass was achieved (Figure 1(b)) [4]. A further percutaneous RFA was carried out two months later, positioning the electrode in an area adjacent to the central necrosis (Figure 1(c)); after this intervention, no tumoral enhancement within the lesion was detected (Figure 2(a)).

The patient subsequently underwent adjuvant radiotherapy with a total amount of 50 Gy fractionated in 25 doses. A complete response was obtained, as shown by a nuclear magnetic resonance (NMR) scan performed 36 months later (Figure 2(b)). A positron emission tomography (PET) scan subsequently confirmed the absence of any metabolic activity within the lesion. Follow-up continued with periodical NMR scans; the last one was performed in May 2012 and it showed stability of the mediastinal picture and a 3.5 cm lesion in the upper lobe of the right lung. A percutaneous CT-guided biopsy allowed the diagnosis of a small cell lung carcinoma. By the end of 2012, the patient was alive and he was exposed to chemotherapy for the carcinoma of the lung.

## 3. Discussion

The current practice in the management of advanced stage thymomas is based on a multidisciplinary approach, including the combination of chemotherapy, surgery, and radiotherapy. Ablative treatments in this setting are only sporadically reported. Yamauchi et al. reported for the first time the use of cryoablation for palliation in a 29-year-old female patient with locally advanced unresectable B3 thymoma [2]. The patient previously underwent chemoradiotherapy. The association with cryoablation produced a significant improvement of her symptoms; she died of lung metastases 41 months later. Zhang et al. recently published their experience with a combination of percutaneous cryotherapy and iodine-125 seed implantation in the treatment of unresectable malignant thymomas. The authors evaluated in 19 cases the safety and effectiveness of the method, as well as its effect on the mass and the progression-free survival (PFS). They concluded that the method is safe and effective, as no major complications occurred; the main body of the lesions was considerably reduced, and the median PFS was 18 months (range 14–29) [3].

The use of radiofrequency thermal ablation is widely reported in the treatment of several thoracic tumors, especially those of the lung, either primitive or metastatic, with or without involvement of the mediastinal lymph nodes. Nevertheless, no reports on its use in the treatment of advanced thymomas have been published; to our knowledge, the present case is the first to be officially reported, as only sporadic unpublished cases have previously been described [5].

The rationale of the RFA use in the treatment of advanced stage thymomas is based on the biological effects of radiofrequency on the neoplastic tissue and on the possibility to accurately caliber the anatomical extension of the treatment. The tissue effects have been extensively described, especially in lung tumors: the neoplastic cells are heated by ionic agitation to over 60° and this causes an irreversible protein denaturation and cell death. Furthermore, RFA is a minimally invasive technique which can be performed in local anesthesia and may be repeated if necessary. Some authors found that the proximity of target lesions to large vessels has been associated with higher local tumor progression rates in patients with lung malignancy [6, 7]. Nevertheless, this may be due to the low thermal conductivity of the lung, which limits heat transfer into pulmonary lesions and prevents ablation with adequate surrounding margins [6, 8]. Thermal conductivity of thymic tissues is greater than that of the lung, allowing a better ablation. These considerations, along with the stage of the tumor, the unfeasibility of a radical surgical resection, and the unresponsiveness to chemotherapy, led our multidisciplinary team to consider and adopt RFA.

Radical resections have been demonstrated to be a significant predictor of survival in patients with thymoma, even when surgery is extended to the adjacent anatomical structures (e.g., pleura, lung, pericardium, and venous vessels). Unfortunately, these conditions only partially represent Masaoka stage III disease, which comprises also unresectable tumors. In these last cases, the only surgical option is an incomplete resection. In terms of survival, the role of debulking in advanced thymomas is still a matter of debate. Some authors have reported an increased 5-year survival rate in up to 30% of cases submitted to incomplete resection of thymomas [1]. Unfortunately, these studies are characterized by a relevant variability of combined approaches, complicating the assessment of the role of the debulking. Furthermore, other reports evidenced no survival advantages to debulking [1]. The possibility to implement incomplete resections with preoperatively planed RFA, as described in our case, may improve results in comparison to other approaches. On the other hand, whether the RFA alone can be an alternative to the debulking should also be assessed.

The multidisciplinary approach used in our case produced excellent results, leading to a complete and stable response. It is difficult to assess which of the single treatments employed had a major impact on obtaining such results. Consequently, it is hard to state that RFA was the “golden bullet.” Nevertheless, we believe that RFA must be taken into account for multidisciplinary treatment of advanced stage thymomas, and its effectiveness must be assessed in the context of adequately designed clinical trials.
